# Massive Splenomegaly and Pancytopenia: It’s a Hairy Situation

**DOI:** 10.7759/cureus.12237

**Published:** 2020-12-23

**Authors:** Sarah M Clark, Farmin Samareh-Jahani, Munir A Chaudhuri

**Affiliations:** 1 Internal Medicine, Emory University School of Medicine, Atlanta, USA

**Keywords:** hairy cell leukemia, massive splenomegaly, pancytopenia, transfusion refractory anemia

## Abstract

A 33-year-old previously healthy Middle Eastern male presented to the emergency department with four weeks of progressively worsening fatigue, dyspnea on exertion, night sweats, and a 10-pound weight loss after suffering a self-limiting viral upper respiratory illness. He was found to be profoundly anemic and thrombocytopenic with normal white blood cell count with a lymphocytic predominance. His anemia was refractory to red blood cell transfusions, to which he developed hyperbilirubinemia. A CT scan revealed hepatomegaly and massive splenomegaly associated with multi-station abdominopelvic lymphadenopathy. A peripheral blood smear revealed several lymphocytes with hairy cell features and bone marrow biopsy revealed hypercellularity with interstitial infiltration by mature lymphoid cells. Flow cytometry confirmed the diagnosis of hairy cell leukemia (HCL) and this patient was initiated on cladribine chemotherapy. This case illustrates the uniqueness of this patient presenting within a short time course, at an atypical age, and with uncommon features for HCL including lymphadenopathy, hepatomegaly, and petechial skin rash. This case also highlights an important point regarding the management of severe anemia in the acute setting while undergoing splenic sequestration. His lack of response to red blood cell transfusions highlights the need for more research on the use of transfusions in patients who are not current surgical candidates for splenectomy.

## Introduction

Hairy cell leukemia (HCL) is a rare form of B-cell lymphoproliferative disorder that accounts for 2% of all leukemias with an incidence in the United States of <0.3/100,000 in men and <0.1/100,000 in women. It is four to five times more common in males and the median age affected is 55 years old [1,2]. Patients may present with infection or other manifestations of cytopenias and/or symptomatic splenomegaly. Laboratory studies can be suggestive of the diagnosis with the characteristic finding of “hairy cells” on the peripheral blood smear. However, bone marrow biopsy is required for definitive diagnosis. In this report, we present the case of a 33-year-old male with hairy cell leukemia diagnosed on bone core biopsy after a dry bone marrow aspiration with atypical features at presentation including age, subacute symptoms, lymphadenopathy, an atypical petechial rash, and normal white blood cell count with lymphocytic predominance [3].

Informed consent was obtained in this study.

## Case presentation

**Figure 1:**
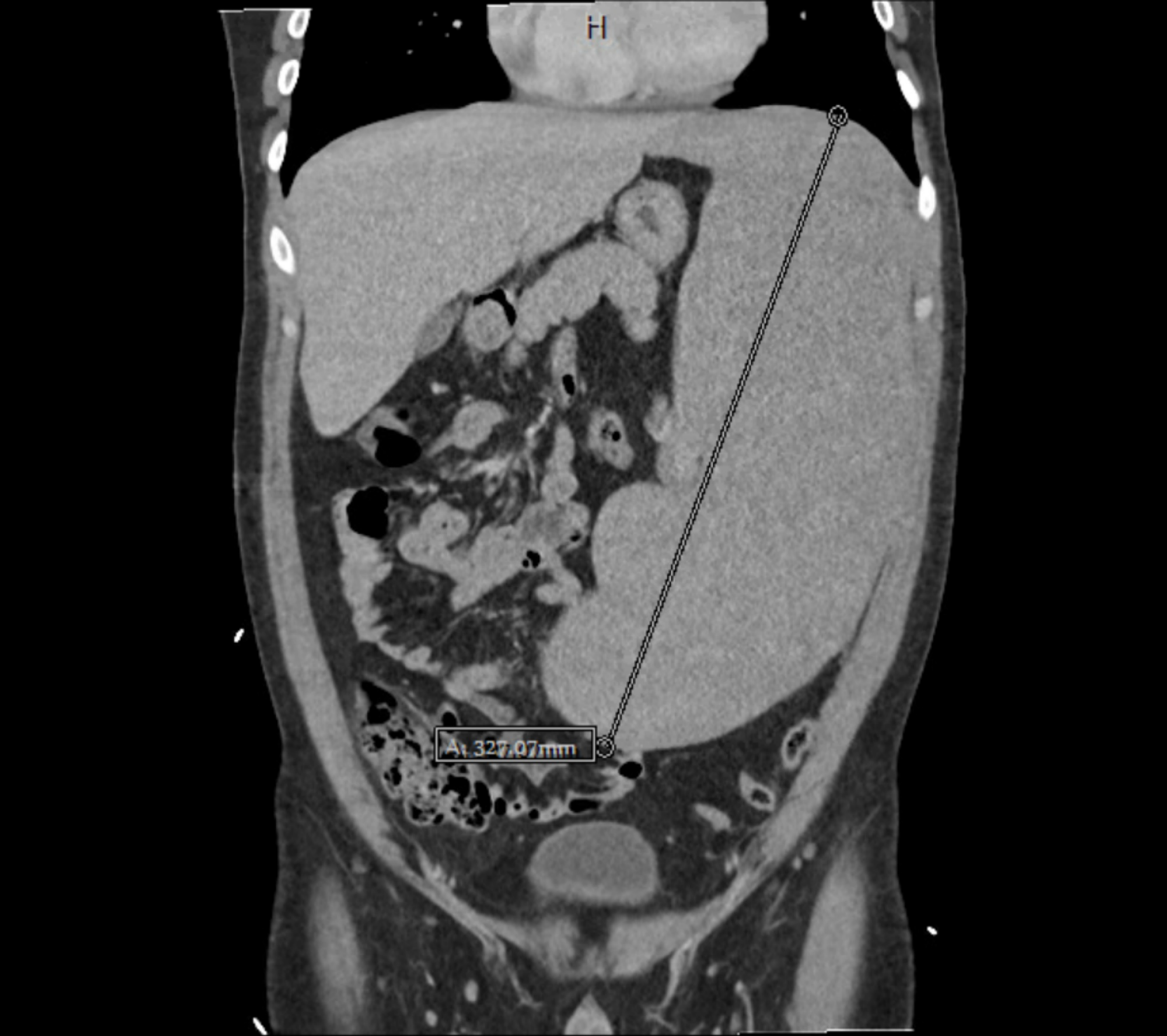
CT abdomen and pelvis with IV contrast demonstrating splenomegaly with largest diameter of 32.7 cm in the craniocaudal direction.

**Figure 2:**
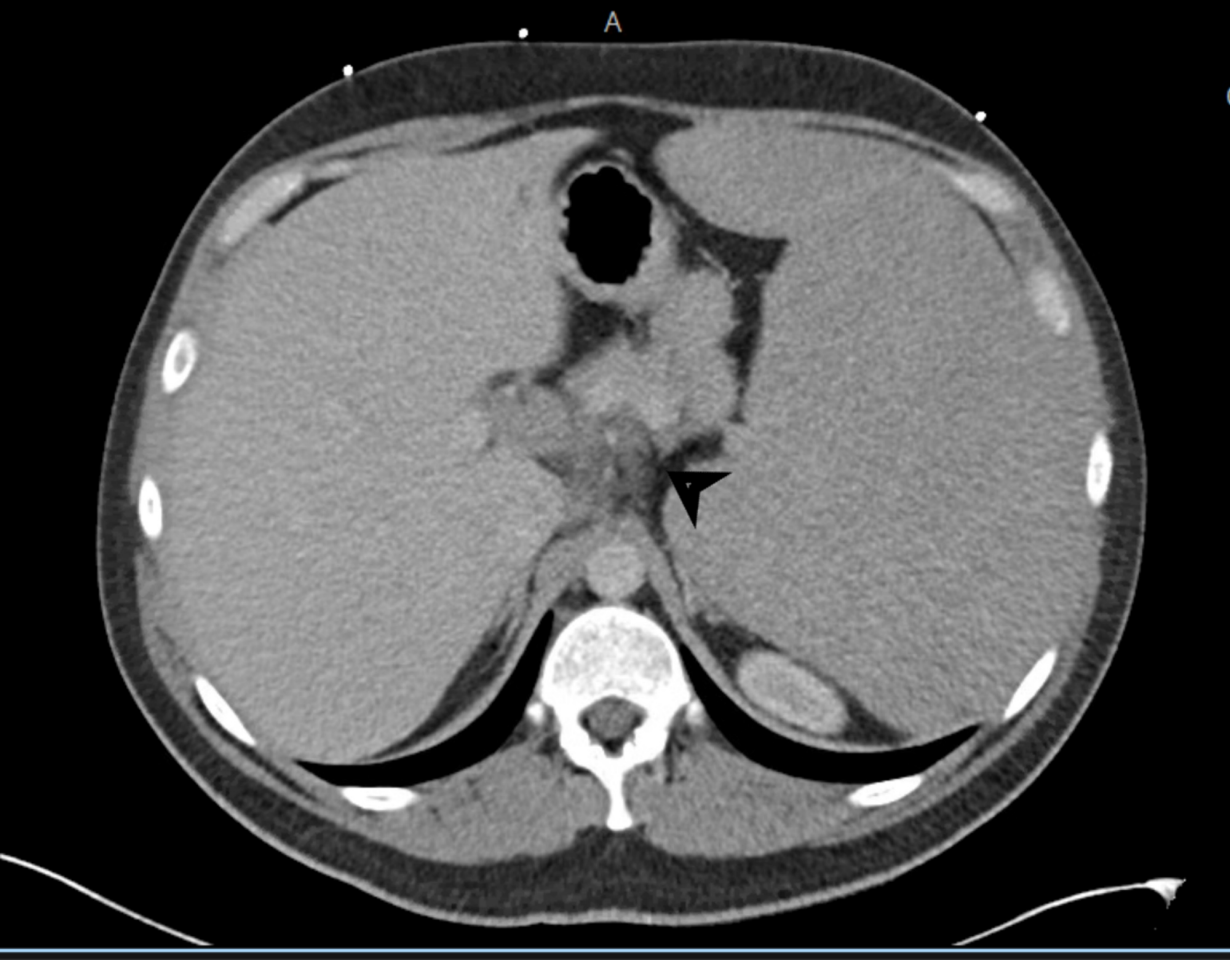
CT abdomen and pelvis with IV contrast demonstrating a 2.6 x 1.4 cm celiac axis lymph node.

**Figure 3:**
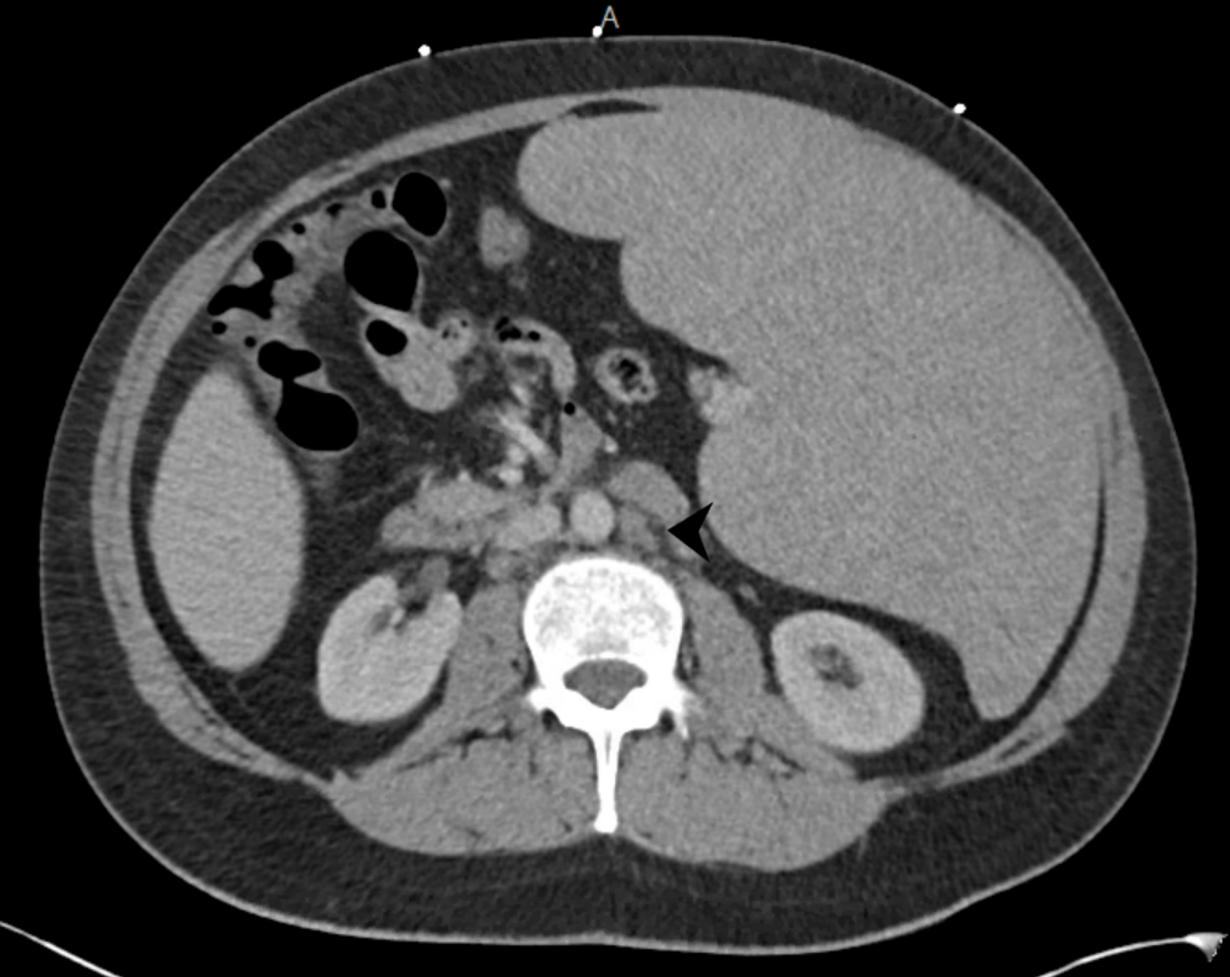
CT abdomen and pelvis with IV contrast demonstrating a 1.4 x 2.5 cm left para-aortic lymph node.

**Figure 4:**
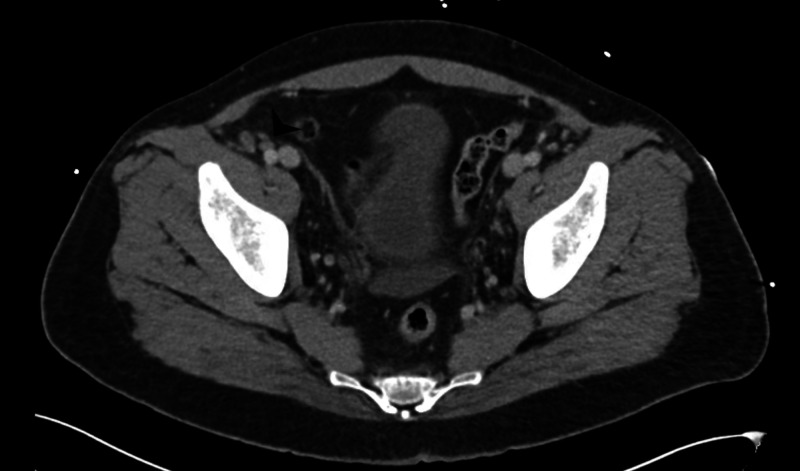
CT abdomen and pelvis with IV contrast demonstrating a 1.3 x 0.8 cm right external iliac lymph node.

A 33-year-old Middle Eastern male with no past medical history presented to the emergency department (ED) with approximately one month of progressively worsening fatigue, shortness of breath, and dyspnea on exertion after having viral upper respiratory infection symptoms around the same time as symptom onset. His roommate had non-laboratory confirmed flu at this time, but he denied exposure to other infectious contacts, travel outside of the United States in the past five years, or recent sexual encounters. He also had a 10-pound unintentional weight loss and generalized sweating during this time period. He endorsed dizziness when transitioning from sitting to standing and reported decreasing blood pressures (BP) measured by home BP cuff, which is ultimately what led him to come to the ED. He was afebrile with a heart rate of 81 beats per minute, respiratory rate of 18 respirations per minute, and blood pressure of 119/72 mmHg. Physical exam was remarkable for pallor, splenomegaly palpated into the right lower quadrant, conjunctival pallor, a petechial rash of the bilateral lower extremities, and bilateral inguinal lymphadenopathy without head or neck lymphadenopathy. On admission, his labs were remarkable for hemoglobin of 4.9 g/dL with a normal MCV and elevated RDW, platelets of 25,000/mcL, and a white cell count of 6,500 with 93% lymphocytes, absolute monocytopenia, and absolute neutrophil count of 420/mcL. The total bilirubin was 1.9 mg/dL. CT imaging of the abdomen and pelvis showed splenomegaly with a maximum diameter of 32.7 cm with multistation lymphadenopathy (see Figures 1, 2, 3, 4).

The initial differential diagnosis for this patient given his age and presentation included viral-induced pancytopenia and a myeloproliferative disorder. Parvovirus, EBV, HIV, Hepatitis B/C, and CMV serologies, however, all returned negative. His peripheral blood smear was reviewed by hematopathology and showed severe anemia, thrombocytopenia, and atypical lymphocytes most consistent with “hairy cell” morphology. Flow cytometry revealed a monotypic B-cell population with CD11c expression. Although the diagnosis was fairly clear at this point, a bone marrow biopsy was collected to make a definitive diagnosis. The bone marrow biopsy resulted in a dry bone marrow aspiration, which is common in HCL [4,5]. A core biopsy showed hypercellular marrow (95%) with extensive involvement by B-cell leukemia and reduced hematopoiesis. Flow cytometry was remarkable for a clonal B-cell population with immunophenotypic features suggestive of hairy cell leukemia (positive for CD11c, CD19, CD20, CD22, CD123, CD200, HLA-DR, and CD45 with lambda immunoglobulin light chain restriction). The cells did not express CD38. These findings further supported the diagnosis of hairy cell leukemia. The sample was later found to be positive for BRAF V600E mutation and IGHV mutation.

Treatment was initially supportive, including two units of packed red blood cells, which only increased his hemoglobin by 0.2 g/dL. He was given multiple blood transfusions throughout his admission and developed an acutely worsening direct hyperbilirubinemia with a maximum total bilirubin of 8.7 mg/dL, likely representing hemolysis of the transfused packed red blood cells.

On day 6 of hospitalization, IV cladribine was initiated and fertility planning prior to chemotherapy was foregone due to the severity of his disease and the need for urgent initiation of treatment. He was also started on neutropenic prophylaxis with acyclovir, fluconazole, and levofloxacin. His platelets and hemoglobin initially dropped after several days of treatment, likely due to a combination of medication side effects and disease pathology. After five days of IV cladribine, he was discharged in stable condition with allopurinol for tumor lysis syndrome prophylaxis and pentamidine for PCP prophylaxis. Pentamidine was selected due to the incidental finding of G6PD deficiency. His counts slowly stabilized and began to recover prior to discharge.

## Discussion

In this case, we describe a 33-year-old male found to have hairy cell leukemia with features suggestive of chronic disease including massive splenomegaly and severe anemia, yet only subacute onset of symptoms after a likely viral upper respiratory infection. Other unique aspects of this patient’s diagnosis are his young age, a normal leukocyte count with lymphocytic predominance, lymphadenopathy, and petechial lesions on his lower extremities. 

Existing literature highlight the uniqueness of this patient’s presentation. The median duration of symptoms ranges from 4 to 6 months, while this patient had symptoms for approximately one month [5,6]. Studies have shown a median age ranging from 47 to 62 years old, although newer data is providing evidence that the true median is likely at the higher end of the range [7-10]. Regarding his leukocyte count of 6,500, one study found only 18.8% of patients had a leukocyte count within the range of 3,500 to 7,000. Other studies reported only 20%-27% of patients presented with two depressed cell lines, while 45%-61.5% showed full pancytopenia [6,7,11]. Additionally, less than 25% of patients had peripheral or abdominal lymphadenopathy on presentation [5,8,12]. To our knowledge, only a small handful of case reports in recent literature have commented on a rash associated with HCL at presentation and prior to diagnosis. Two articles reported a leukocytoclastic/lymphocytic vasculitic rash, while one reported concomitant Sweet syndrome [13-15]. One study from 1984 reviewed cutaneous findings associated with HCL in 113 patients. However, many of the cutaneous lesions were infectious or manifestations of cytopenias (e.g., ecchymoses, pallor), differentiating these cutaneous findings from the lesions in the patient discussed in this report [16]. Another study found an association between vasculitis and HCL, however, the signs and symptoms of vasculitis presented only after the diagnosis of HCL had already been made [17]. Of note, one study concluded that vasculitis may be in direct relation with HCL as evidenced by the resolution of vasculitis with treatment for HCL [12]. 

The definitive diagnosis of HCL is based on bone marrow biopsy with immunophenotypic features including expression of CD19, CD20, CD22 and CD200 and positive CD11c, CD103, CD123 and CD25 markers. 98% of HCL cases express 3-4 of the latter four listed markers, while this patient only expressed two. BRAF V600E mutation is identified in 70-100% of patients diagnosed with HCL [3]. In this patient, a dry bone marrow aspiration prevented the analysis of bone marrow biopsy, but immunophenotypic features of the peripheral blood smear were consistent with HCL, including BRAF V600E mutation. This highlights the utility of using samples beyond the gold standard of bone marrow biopsy to make the diagnosis of HCL. 

The prognosis for patients with HCL is generally quite good with normal life expectancy in patients who have complete or partial remission after receiving first-line therapies, although mortality is increased in younger subsets of patients [18]. Similar to chronic lymphocytic leukemia, unmutated immunoglobulin heavy chain variants (IGHV) portends worse prognosis and decreased response to current treatments. Multicenter data shows bulky spleen and leukocytosis were found to be predictors of treatment failure in addition to single-center data suggesting lymphadenopathy and leukopenia as additional poor prognostic factors. Fortunately, up to 40% of patients with splenomegaly or leukocytosis will still respond to first-line treatments [19]. Lack of BRAF V600E mutation has also been associated with worse prognosis and occurs in 10-20% of HCL cases [3]. 

Additional important considerations include the utility of blood transfusions in patients with anemia in the setting of HCL. Non-sickle cell-related splenomegaly and its effect on red blood cell sequestration and transfusion outcomes are not well described in the literature. A published case report describes a patient with hematologic malignancy who responded poorly to blood transfusion in the setting of splenomegaly and without evidence of immune-mediated hemolysis. The patient was treated with splenectomy, which stabilized and returned his hemoglobin to near normal even five months after surgery [20]. Our case illustrates and further supports that in a patient with massive splenomegaly and chronic anemia, even if severe, transfusion may only be useful if the patient is actively bleeding. This patient provides further evidence of a massive spleen‘s ability to sequester large amounts of blood and induce non-immune mediated hemolysis while putting neutropenic patients at risk for infection if using nonradiated blood products. This is especially relevant in patients who have not yet received a diagnosis of HCL. This is evidenced by the acutely worsening hyperbilirubinemia secondary to hemolysis that could be correlated with the timing of blood transfusions. In this patient with severe anemia and splenomegaly, blood transfusions may have had a detrimental impact on an otherwise stable patient secondary to hemolysis. It is important to consider the differential diagnosis for anemia and massive splenomegaly which includes, but is not limited to, leukemias (chronic myeloid leukemia, chronic lymphocytic leukemia, hairy cell leukemia), non-Hodgkin lymphoma, myelofibrosis, metastatic cancer (of the bone marrow or spleen), primary tumors of the spleen (vascular, lymphoid, and non-lymphoid), infection (e.g. malaria, leishmaniasis, schistosomiasis, typhoid fever, mycobacterium), autoimmune hemolytic anemia, β-thalassemia major, megaloblastic anemia, hereditary spherocytosis, and infiltrative conditions (sarcoidosis, Gaucher disease, Niemann-Pick disease, hemophagocytic lymphohistiocytosis). Further research needs to be conducted to examine the risks and benefits of blood transfusions in the patient with anemia and splenomegaly who is not hemorrhaging and does not have a history of sickle cell disease.

## Conclusions

This case highlights the heterogeneity of presentations in patients with HCL, including young age, subacute symptoms, normal white blood cell count with lymphocytic predominance, lymphadenopathy, and a petechial rash. Although splenomegaly is a known feature of HCL, it is important to consider this diagnosis if massive splenomegaly is found on exam or imaging. This case also emphasizes the ability to utilize a peripheral blood smear sample to evaluate for immunophenotyping, which could be done immediately upon dry bone marrow aspiration while waiting for core biopsy results. Additionally, this patient’s response to blood transfusions with elevated bilirubin and minimal increase in hemoglobin suggests it would be beneficial to study patients with splenomegaly secondary to causes unrelated to sickle cell disease. This is especially relevant for a patient such as the one presented here who is not a current surgical candidate for splenectomy due to thrombocytopenia and high risk for bleeding.
